# Scene Construction and Spatial Processing in Post-traumatic Stress Disorder

**DOI:** 10.3389/fnbeh.2022.888358

**Published:** 2022-06-29

**Authors:** Hannah Marlatte, Derek Beaton, Sarah Adler-Luzon, Lina Abo-Ahmad, Asaf Gilboa

**Affiliations:** ^1^Department of Psychology, University of Toronto, Toronto, ON, Canada; ^2^Rotman Research Institute, Baycrest Health Sciences, Toronto, ON, Canada; ^3^Data Science and Advanced Analytics, St. Michael’s Hospital, Unity Health Toronto, Toronto, ON, Canada; ^4^Department of Psychology, University of Haifa, Haifa, Israel

**Keywords:** scene construction, post-traumatic stress disorder (PTSD), spatial processing, hippocampus, cingulum bundle

## Abstract

**Introduction:**

Post-traumatic stress disorder (PTSD) is associated with hippocampal system structural and functional impairments. Neurobiological models of PTSD posit that contextual memory for traumatic events is impaired due to hippocampal system dysfunction whilst memory of sensory details is enhanced due to amygdalar impact on sensory cortices. If hippocampal system dysfunction is a core feature of PTSD, then non-traumatic hippocampal-dependent cognitive functions such as scene construction, spatial processing, and memory should also be impaired in individuals with PTSD.

**Methods:**

Forty-six trauma survivors, half diagnosed with PTSD, performed two tasks that involved spatial processing. The first was a scene construction task which requires conjuring-up spatially coherent multimodal scenarios, completed by all participants. Twenty-six participants (PTSD: *n* = 13) also completed a navigation task in a virtual environment, and underwent structural T1, T2 and diffusion-tensor MRI to quantify gray and white matter integrity. We examined the relationship between spatial processing, neural integrity, and symptom severity in a multiple factor analysis.

**Results:**

Overall, patients with PTSD showed impaired performance in both tasks compared to controls. Scenes imagined by patients were less vivid, less detailed, and generated less sense of presence; importantly they had disproportionally reduced spatial coherence between details. Patients also made more errors during virtual navigation. Two components of the multiple factor analysis captured group differences. The first component explained 25% of the shared variance: participants that constructed less spatially coherent scenes also made more navigation errors and had reduced white matter integrity to long association tracts and tracts connecting the hippocampus, thalamus, and cingulate. The second component explained 20% of the variance: participants who generated fewer scene details, with less spatial coherence between them, had smaller hippocampal, parahippocampal and isthmus cingulate volumes. These participants also had increased white matter integrity to the right hippocampal cingulum bundle.

**Conclusion:**

Our results suggest that patients with PTSD are impaired at imagining even neutral spatially coherent scenes and navigating through a complex spatial environment. Patients that showed reduced spatial processing more broadly had reduced hippocampal systems volumes and abnormal white matter integrity to tracts implicated in multisensory integration.

## Introduction

Memory aberrations are central to post-traumatic stress disorder (PTSD) clinical phenomenology. One hallmark of the disorder is enhanced involuntary memories, including intrusive re-experiencing of the trauma as happening in the present that is sensory-detailed and vivid (Van der Kolk and Fisler, 1995; Frewen and Lanius, 2006; Brewin, 2007). Paradoxically, at the same time patients suffer an impaired ability to voluntarily retrieve traumatic and non-traumatic memories. Voluntary remembering of traumatic memories is characterized as being fragmented and disorganized (Frewen and Lanius, 2006; Brewin, 2007; Dalenberg et al., 2007) and this memory incoherence was found to be predictive of the disorder’s severity at later stages (Jones et al., 2007; but see: Porter and Birt, 2001 and Rubin et al., 2008).

The mnemonic symptomatology of PTSD is thought to result from disorder-related changes to the hippocampus. The hippocampus is the epicenter of neural networks that support a myriad of cognitive processes that involve binding information and representing relationships across space and time, including memory. Reduced hippocampal volume has frequently been reported in association with chronic and severe PTSD (Shin et al., 2006; Logue et al., 2018) and hippocampal abnormalities may be a vulnerability factor to developing more chronic severe forms of the disorder (Gilbertson et al., 2002; Woon et al., 2010; van Rooij et al., 2015). Beyond the hippocampus, PTSD patients sometimes display abnormalities in other limbic and paralimbic neural structures, including smaller amygdala (Karl et al., 2006; Shin et al., 2006; Logue et al., 2018) and smaller anterior cingulate cortex (Karl et al., 2006) which may be acquired post-trauma (Kasai et al., 2008). These structural differences are also related to abnormal function in the hippocampus, amygdala, and frontal regions (Rauch et al., 2006). Such findings have inspired neurobiological models that emphasize the centrality of declarative memory impairments as the basis for the development and persistence of PTSD (e.g., Elzinga and Bremner, 2002; Rauch et al., 2006).

Despite pervasive findings of hippocampal abnormalities in PTSD, evidence is mixed for more generalized hippocampally-mediated cognitive deficits outside of the traumatic memories themselves. Medium sized memory impairments on neuropsychological and experimental tests of memory have been reported (e.g., Lambert and McLaughlin, 2019). However, often these effects are either diluted or disappear when contributing factors mediated by prefrontal-subcortical circuits such as attentional and working-memory deficits are considered (e.g., Isaac et al., 2006; Gilboa, 2015; Scott et al., 2015). Indeed, deficits are common in PTSD in the domains of attention, working memory and executive functions that could lead to secondary deficits on tests of long-term memory (Horner and Hamner, 2002; Isaac et al., 2006; Qureshi et al., 2011; Gilboa, 2015). Evidence is also mixed for a relationship between memory impairments, hippocampal structure and function, and PTSD symptomatology (Tischler et al., 2006; Chen et al., 2009; Woodward et al., 2009).

There is, however, more consistent evidence for deficient autobiographical memory in individuals with PTSD. When asked to recall specific autobiographical memories, people suffering from severe trauma display over-general personal memories and have difficulty retrieving specific instances (McNally et al., 1995; McNally, 1998), a trait that may be predictive of developing the disorder (Bryant et al., 2007) and is likely independent of the valence of the cues or memories (Sutherland and Bryant, 2008). Impoverished autobiographical memory may also extend to imagined future events and be specific to internal, episodic details (Brown et al., 2013; reanalyzed in Brown et al., 2014). Spatial processing is another cognitive domain that significantly depends on hippocampal function for intact performance. Deficits have been noted in patients with PTSD specifically for aspects of spatial processing that are hippocampal-dependent, like allocentric spatial processing which involves representing the relationships among environmental features (Gilbertson et al., 2007; Smith et al., 2015). Such deficits have been found in PTSD patients even when controlling for general visuospatial ability and may be a genetic predisposing factor, as the non-traumatized identical twins of patients with PTSD also showed impairment (Gilbertson et al., 2007).

The apparent gap between the rather dramatic memory-related clinical phenomenology of PTSD, consistent findings of abnormal hippocampal structure and function, but only mixed evidence for general memory deficits may be related to how episodic memory is tested. This possibility aligns with the more consistent deficits in the autobiographical and spatial processing domains described above. One recent idea is that the core function of the hippocampus is scene construction, a process that underpins several related cognitive functions including episodic memory, navigation, and imagination (Hassabis and Maguire, 2007). Scene construction requires mentally generating and maintaining complex and coherent scenes or events by retrieving and integrating relevant sensory details from modality-specific cortical areas. This process leads to representations with a coherent spatial context that can be manipulated and visualized. This conception of hippocampal function corresponds well with the phenomenology of PTSD and could explain the existence of memory fragments devoid of their context, lacking spatial coherence and integration. This is also consistent with dual-representation model of PTSD where sensory details of traumatic memories are encoded strongly as sensory representations but contextual details are tenuously encoded as their own representations (Brewin et al., 2010). Sensory details are therefore not well integrated into the appropriate context, which may explain why flashback memories are inflexible, sensory, and vividly experienced in the present, but can also contain significant narrative gaps.

This neurobiological theory suggests that it is information transfer and integration that is awry in PTSD. Based on that, one would expect that in addition to gray matter differences in patients with PTSD there will also be abnormal white matter connectivity between structures, most notably in the limbic system which includes the hippocampus. However, only a handful of studies have explored white matter integrity differences in PTSD patients, with inconsistent findings across individual studies. Two recent meta-analyses have found decreases to fractional anisotropy (FA) in the cingulum (Daniels et al., 2013; Ju et al., 2020), the most prominent white matter tract in the limbic system. Changes to long association tracts have also been found, with notable increases in FA found to the inferior fronto-occipital fasciculus (IFOF) connecting regions across occipital, temporal, parietal and frontal lobes (Ju et al., 2020). Thus far, there is little research on how white matter structural abnormalities in the brain relate to behavior in PTSD. We wanted to explore scene construction ability in traumatized individuals with and without PTSD, and explore how scene construction ability relates to neural integrity in both gray matter volume and white matter tract integrity in a subset of participants. This subset also completed a navigation task to allow us to also assess spatial processing broadly.

To our knowledge, this is the first study to assess scene construction in PTSD and to explore the relations of spatial processing to neural integrity using both gray matter volume and white matter integrity.

## Materials and Methods

### Participants

Forty-six trauma survivors participated in the study; half developed chronic PTSD and half did not and were thus trauma-exposed matched controls. Participants with PTSD were recruited from a clinic in Haifa and diagnosed by a staff psychiatrist. Most patients’ criterion A event was a warfare event (missile attacks) encountered as civilians (*n* = 12) or as reservists on active duty (*n* = 4). Other events included traffic accidents (*n* = 3), work accidents (*n* = 2) and stabbing/terror attacks (*n* = 2). Matched controls were recruited through a snow-ball method from fellow participants. They were typically present at the same or similar events as the patients but did not develop PTSD. Events included missile attacks as civilians (*n* = 14) or as reservists (*n* = 7) and traffic accidents (*n* = 2). Controls were also well matched for age, sex, and education, but significantly differed from patients on clinical measures of PTSD, anxiety, and depression. See Table 1 for a demographic and clinical summary and Table 2 for assessments of cognitive function.

**TABLE 1:** Demographic and clinical summary.

	PTSD M (SD)	Controls M (SD)	*t*
N (Female)	23 (10)	23 (10)	
Age	42.70 (11.32)	38.43 (12.67)	1.20
Education	12.83 (1.85)	13.70 (2.29)	−1.42
CAPS	87.26 (22.86)	5.04 (5.95)	16.69***
LEI	6.04 (6.70)	2.04 (1.30)	2.81**
STAI–State	63.65 (18.31)	38.13 (11.72)	5.63***
STAI–Trait	69.22 (14.40)	43.43 (11.86)	6.63***
BDI-II	24.70 (9.67)	5.04 (4.67)	8.78***

**p < 0.01, ***p < 0.001.

**TABLE 2:** Assessment of cognitive function.

	PTSD	Controls	*t*
**Verbal fluency**			
Semantic (z-score)	−0.24 (1.36)	0.20 (0.98)	1.18
Phonemic (z-score)	−0.08 (0.98)	−0.93 (0.89)	2.91**
**Digit Span (scaled score)**	8.48 (2.21)	9.39 (2.25)	1.39
**Shipley-2**			
Vocabulary	27.69 (10.30)	34.00 (6.39)	1.88
Abstraction	18.77 (9.98)	30.15 (7.37)	3.31**
Estimated IQ	91.15 (16.83)	108.23 (12.69)	2.92**

**p < 0.01.

All participants completed a scene construction task where they imagined and described novel scenes. A subset of participants, 13 controls and 13 patients, also completed a navigation task in a virtual environment and underwent T1/T2 and diffusion tensor MRI to assess gray and white matter integrity.

### Procedure

All participants provided informed consent. To determine the current severity of PTSD symptoms, participants first completed a clinical assessment of symptom severity using the Clinician-Administered PTSD Scale (CAPS; Blake et al., 1995). Participants were also assessed on their history of past trauma through the Lifetime Event Inventory (LEI), their degree of state and trait anxiety with the State-Trait Anxiety Inventory (STAI), and their degree of depression with the Beck Depression Inventory (BDI-II). Basic cognitive function was assessed using the verbal fluency test, the digit-span test, and the Shipley-II measure of static and fluid intelligence which provides an estimation of IQ.

#### Scene Construction Task

Participants were assessed on their scene construction ability using the task developed by Hassabis et al. (2007). Participants were instructed to vividly imagine then describe a series of ordinary, common-place scenes consisting of seven fictitious scenarios and two personal future events. One scenario from the original protocol was removed as it involved constructing a narrative rather than a single event. Each participant was tested individually and faced the interviewer, who read aloud each scenario from a prepared script and provided prompts to aid in detail generation. Participants’ narratives were recorded and later transcribed for scoring.

For each scenario, participants were asked to describe each imagined scene in as much multimodal detail as possible (for example, “Imagine you are lying on a white sandy beach in a beautiful tropical bay. I want you to describe the experience and the surroundings in as much detail as possible using all of your senses including what you can see, hear and feel”). Participants were explicitly asked not to recall an actual memory but create something new and continue with their descriptions until they came to a natural end or felt like nothing else could be added. After each scenario, participants provided subjective ratings of salience and presence within the scene and completed a questionnaire regarding the spatial coherence. Participants rated their sense of presence within the scene and their perceived salience on scales from 1 to 5 (see Supplementary Material 1.1). The spatial coherence index assessed how integrated details were within the greater scene, where each participant was presented with a list of 12 statements from which they chose all the ones that best described their constructed scene (see Supplementary Material 1.2). Eight of the statements describe an integrated and continuous experience (e.g., “I could see it as one whole scene in my mind’s eye”), and four described a fragmented one (e.g., “It wasn’t so much a scene as a collection of images”). This provided a score ranging from −4 to 8 that was normalized around zero to range from −6 (totally fragmented) to 6 (completely integrated).

An external scorer later assessed each transcribed narrative based on scene content and scene quality. To assess scene content, transcripts were first segmented into informational bits and the content was classified according to the manual developed by Hassabis et al. (2007) to one of four categories: a spatial reference (SPA), an entity present (EP), a sensory description (SD), or a thought, emotion, or action (TEA). For each category there was a limit of seven statements per scene so that the maximum possible content score per scene was 28 (see Supplementary Material 1.3 for scoring samples). External scorers also judged the overall quality of the constructions based on how detailed a picture of the experience was evoked in the scorer’s mind based on the transcripts alone ranging from 0 to 10 (see Supplementary Material 1.4).

Finally, a composite score called the Experiential Index was calculated to measure the overall richness of the imagined scene using normalized scores of objective scene content, subjective ratings of scene quality and spatial coherence by the participant, and quality judgments by the scorer. To obtain such a score, participants’ ratings of salience and presence were rescaled from 1–5 to 0–4, negative spatial coherence ratings were rounded to zero, and the quality judgment scores were rescaled to 0–18. The Experiential Index ranged from 0 to 60.

#### Virtual Navigation Task

**FIGURE 1:**
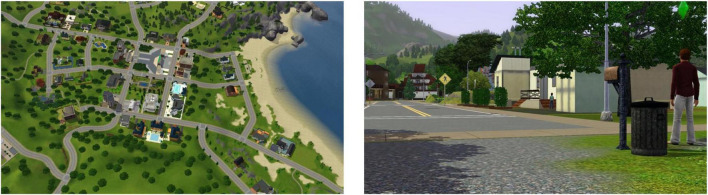
Navigation memory task. Left: Bird’s eye view of the virtual town. Right: Starting point view as seen by participants. Sim City and screenshots of it are licensed property of Electronic Arts, Inc. Reproduced with permission.

Twenty-six participants–half diagnosed with PTSD–also completed a virtual navigation test in an environment created using the SimCity video game platform (Figure 1). Participants were told they would be shown a particular route with several turns and landmarks along the way and that they were to try and remember the route for a later test. They first passively viewed in first-person a 6-min route at walking pace in a virtual town on a computer monitor that included 13 turns and eight landmarks (e.g., town hall, corner store, swimming pool, diner etc.). As they travelled the route, they heard the names of the different landmarks as well as the travel instructions (e.g., “turn left at town hall”). Next, participants traveled the same route by following arrows on the road by pressing the arrow keys on a keyboard. This phase required participants to actively follow the route but still allowed for errorless learning of the layout and still included the verbal instructions. Finally, participants were asked to use the arrow keys to actively navigate the virtual environment in first-person to reproduce the route, but this time without any guidance. If a participant made an error such that they turned the wrong direction, they were corrected in space and then allowed to continue navigating the route.

Participants were scored on the number of correct turns made during the final active navigation phase as a measure of navigation performance. Additionally, participants performed a vector mapping task where they saw four pictures of landmarks and were told that they were standing in one of them. They had to indicate the direction of the two other landmarks on a blank outline of the map for a total of eight points.

#### Neuroimaging

The same subset of participants who completed the virtual navigation task also underwent structural and diffusion tensor MRI to assess anatomical differences. Participants were scanned using a 3T GE scanner (Signa MR750; GE Medical Systems, Milwaukee, WI, United States) at Rambam Health Care Campus, in Haifa, Israel. Anatomical 3D sequence spoiled gradient (SPGR) echo sequences were used to obtain high-resolution 1 mm × 1 mm × 1 mm voxel size structural MRI images (matrix: 256 × 256; TR/TE = 8/3.1 ms). DTI were acquired with whole-brain voxel resolution of 2 mm × 2 mm × 2 mm (TR/TE = 8,000/85.4; FA = 90°, Δ/δ = 33/26, b = 1,000 s/mm2) with 25 gradient directions and five additional images with no diffusion weighting (B0 image).

Anatomical scans were analyzed using the FreeSurfer neuroimaging suite. The DICOM image files were first converted into FreeSurfer’s MGZ format through the neuroimaging software suite, which were then used by FreeSurfer for cortical reconstruction using the standard reconstruction steps (Fischl, 2012). Briefly, the reconstruction scripts automatically performed anatomical conformation, intensity normalization, skull stripping, subcortical segmentation and labeling, surface reconstruction and (spherical) registration, cortical parcelation, and generation of statistics files containing measurements including volume, area, and thickness. Following automated reconstruction, the 2D volume slices and 3D surfaces were visually inspected using FreeSurfer’s tkmedit and tksurfer packages to identify any visible deviations of the algorithm at the individual anatomy level.

DTI data were reformatted into NIFTI from its original DICOM format using the FMRIB Software Library (FSL). The “eddy_correct” function in FSL was used for motion and for eddy currents corrections using affine registration to b0. No participant had movement in excess of 3 mm translation or two degrees rotation and so all participants were included in the analyses. We used a fractional intensity threshold of 0.3 to create a brain mask which was applied to analyses of diffusion tensors. FSL’s dtifit tool was used for linear fitting of diffusion tensors to the DWI images and maps of DTI scalars were obtained including fractional anisotropy (FA), mean diffusivity, axial diffusivity, and radial diffusivity. We used the Tract-Based Spatial Statistics (TBSS) tool in FSL to create a study-specific FA skeleton by non-linear alignment of individual participants’ FA maps to the FSL template FMRIB58 FA map in MNI 152 standard space. A threshold of FA > 0.2 was used to create the mean FA skeleton, reflecting white matter tracts common across participants. The same process was applied to the other DTI scalars. In the present report we focus on the FA scalar because it is highly sensitive to microstructural integrity across white matter tracts.

#### Statistical Analyses

##### Univariate

Assessment of performance on the scene construction task began by comparing the two groups on the composite Experiential Index score using an independent-sample *t-*test. This was followed by a series of *t-*tests comparing specific aspects of the narratives including total number of details imagined, spatial coherence of details within the imagined scenes, presence within scenes, and saliency of the imagined scenes. Two 2 × 4 between-subjects ANOVAs were also conducted to examine potential differences in the types of details imagined, and if there was an interaction between the type of scene details imagined and PTSD diagnosis. One ANOVA examined detail count and the other the proportion of details imagined. There were planned contrasts for exploring the relationship between group membership and each detail type. Performance on the virtual navigation task was assessed using a 2 × 2 between-subjects ANOVA, with planned contrasts including two independent-samples *t*-tests between groups: one comparing navigation performance and another comparing vector mapping performance.

##### Multivariate

To explore how these two hippocampal-dependent processes of scene construction and virtual navigation are related to neural integrity, a multiple factor analysis (MFA) was conducted for the same subset of participants. MFA is an extension of principal component analysis that is tailored to handle multiple data tables that measure different sets of variables, all collected on the same observations. MFA–like PCA–is a latent variable modeling approach that explains the covariance between a set of observed variables by a set of fewer unobserved (or latent) variables called components. Variables are chosen to be included in the analysis specifically to reflect a hypothesized model of these latent relationships. Tests of permutation are used to identify which components to retain (i.e., those that significantly differ from a null distribution), and bootstrapping are used to identify which variables significantly contribute to each component. Variables that significantly contribute to a latent component covary with each other, either negatively or positively, which can provide insight to complex relationships whilst retaining statistical power.

The MFA was performed using the ExPosition package in R (Beaton et al., 2014) and included four sets of data: gray matter regional volumes, white matter tracts FA, scene construction performance, and virtual navigation performance (see Supplementary Table 1 for a full list of variables). Gray matter volumes and white matter tracts were selected for the multivariate analysis based on their reported involvement in top-down and bottom-up aspects of spatial cognition, imagery, and multisensory integration (Whittingstall et al., 2014; Li et al., 2019; Spagna et al., 2021). All variables were controlled for age, education, and sex using a regression analysis. Gray matter volumes were first calculated as proportional to the individuals whole brain volume, and white matter tracts FA were also controlled for ventricle volume differences. Permutation based resampling methods were used to infer the significance of each component and bootstrapping was used to infer the significant contributions of each variable at *p* < 0.05. Of note, group membership was not explicitly defined *a priori*. Group contributions were explored through visualizations in MFA and by correlating participants’ individual contribution to significant components with their symptom severity using the non-parametric Kendall’s tau.

## Results

### Scene Construction Task

**FIGURE 2:**
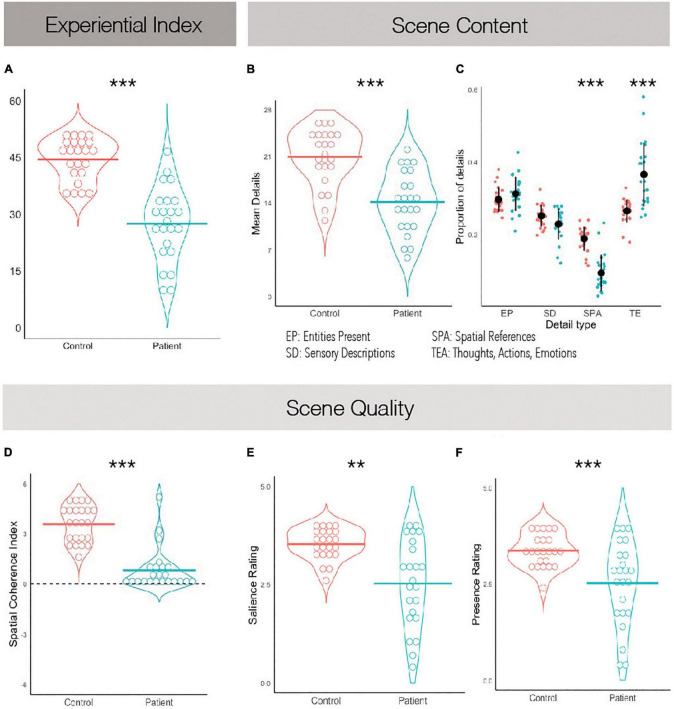
Scene construction task performance. **(A)** The experiential index is a composite score summarizing scene construction ability broadly. **(B)** The mean detail count averaged across imagined scenes. **(C)** The average proportion of details imagined grouped by what kind they are. **(D)** The spatial coherence index, summarizing how integrated the details were within the greater imagined scene. A score of +6 suggests a completely integrated scene and a score of −6 suggests a completely fragmented one. **(E)** Average subjective ratings of salience. **(F)** Average subjective ratings of presence. **p < 0.01, ***p < 0.001.

A Welch’s *t*-test revealed a significant and large effect of group on the composite experiential index of imagined scenes, *t*(34.5) = 7.29, *p* < 0.001, Cohen’s *d* = 2.15, such that scenes imagined by PTSD patients were overall less rich compared to scenes imagined by controls (Figure 2A; see Table 3 for descriptive statistics).

**TABLE 3:** Performance on the scene construction task.

	PTSD	Controls	*t*
	M (SD)	M (SD)	
Overall Experiential Index	27.44 (9.81)	44.52 (5.47)	7.29***
**Scored Content (Transcripts)**			
Total Details	14.17 (4.64)	20.94 (4.22)	5.18***
Spatial References	1.47 (1.21)	4.00 (1.28)	6.91***
Entities Present	4.42 (1.50)	6.12 (1.03)	4.49***
Sensory Descriptions	3.37 (1.46)	5.23 (1.14)	4.82***
Thoughts, Emotions, Actions	4.91 (1.20)	5.58 (1.35)	1.17
**Subjective ratings**			
Presence (1–5 Likert)	2.53 (1.08)	3.37 (0.44)	3.46**
Salience (1–5 Likert)	2.53 (1.10)	3.53 (0.39)	4.11***
Scorer Rating (0–10 Likert)	4.10 (2.19)	7.27 (0.90)	6.44***
Spatial Coherence Index	0.83 (1.27)	3.59 (1.08)	7.92***

**p < 0.01, ***p < 0.001.

Comparing the mean number of details within imagined scenes as reported by participants, it was found that patients described fewer details overall than controls, *t*(44) = 5.18, *p* < 0.001, Cohen’s *d* = 1.53 (Figure 2B). We then examined whether different types of details were equally impoverished in the patients’ narratives. A between-subjects ANOVA revealed a significant effect of scene detail type, *F*(3,132) = 118.90, *p* < 0.001, η2 = 0.31, and a significant interaction of scene detail type and group, *F*(3,132) = 12.48, *p* < 0.001, η2 = 0.03. PTSD patients imagined fewer entities within the scenes than controls, *t*(44) = 4.49, *p* < 0.001, η2 = 1.32, along with fewer sensory descriptions, *t*(44) = 4.82, *p* < 0.001, η2 = 1.42, and fewer spatial details, *t*(44) = 6.91, *p* < 0.001, η2 = 2.04. No differences between groups were noted when comparing the number of person-related details imagined, which includes thoughts, emotions and actions, *t*(44) = 1.77, *p* = 0.08, η2 = 0.52. To further explore this while controlling for the overall impoverished narratives of PTSD patients, we calculated the proportion of details described of each type (Figure 2C). A between-subjects ANOVA again revealed a significant effect of scene detail type, *F*(3,132) = 95.1, *p* < 0.001, η2 = 0.68, and a significant interaction of scene detail type and group, *F*(3,132) = 24.7, *p* < 0.001, η2 = 0.36. *Post hoc* tests revealed that PTSD patients and controls did not significantly differ on the proportion of entities in the imagined scene, *t*(132) = 1.14, *p* = 0.947, or the proportion of sensory descriptions, *t*(132) = −1.59, *p* = 0.753. However, PTSD patients reported a significantly greater proportion of person-related details than controls, *t*(132) = 7.12, *p* < 0.001, and a significantly smaller proportion of spatial details, *t*(132) = −6.66, *p* < 0.001. This suggests that overall PTSD patients imagined less spatially coherent scenes than controls, and perhaps were compensating for a deficit in spatial detail generation with additional person-related details.

Regarding subjective ratings of experienced scene quality, Welch’s *t*-tests revealed an effect of group on subjective presence, *t*(29) = 3.46, *p* = 0.002, Cohen’s *d* = 1.02, and subjective salience, *t*(27.4) = 4.11, *p* < 0.001, Cohen’s *d* = 1.21, such that PTSD patients felt less present within the scenes they imagined and experienced their imagined scenes as less vivid than those imagined by controls (Figures 2E,F). An independent-samples *t*-test also revealed an effect of group on the spatial coherence questionnaire, *t*(44) = 7.92, *p* < 0.001, Cohen’s *d* = 2.34, such that scenes imagined by PTSD patients were less integrated than those imagined by controls (Figure 2D). Finally, Welch’s *t-*test revealed an effect of group on scene quality as rated by an external scorer, *t*(29.3) = 6.44, *p* < 0.001, Cohen’s *d* = 1.90, such that scenes imagined by PTSD patients had overall reduced quality than controls. These were all large effect sizes.

Overall, these results suggest that PTSD patients are impaired at scene construction with preferential impairment of spatial aspects of the task.

### Virtual Navigation Task

**FIGURE 3:**
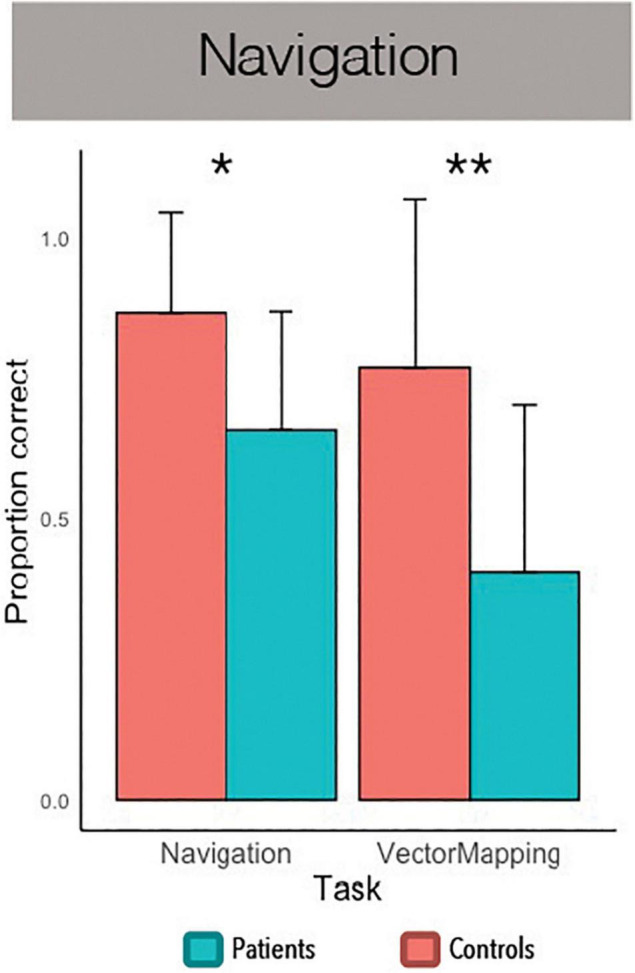
Navigation task performance. *p < 0.05, **p < 0.01.

A subset of twenty-six participants, half with PTSD and half without, also performed a navigation task. A between-subjects ANOVA revealed a significant effect of navigational spatial processing, *F*(1, 24) = 26.18, *p* < 0.001, η2 = 0.52, and a significant interaction of navigational spatial processing and group, *F*(1, 24) = 5.43, *p* = 0.029, η2 = 0.19. An independent-samples *t-*test revealed an effect of group on virtual navigation, *t*(24) = 2.70, *p* = 0.012, Cohen’s *d* = 1.06, such that controls made fewer navigation errors than patients (see Figure 3). Similarly, an independent-samples *t-*test revealed an effect of group on vector mapping, *t*(24) = 3.13, *p* = 0.005, Cohen’s *d* = 1.23, such that controls more accurately estimated the distance and direction of landmarks in the virtual town than patients. Although the number of participants per group was modest, these were both large effects, suggesting that PTSD patients are robustly impaired at navigational spatial processing.

### Multiple Factor Analysis

**FIGURE 4:**
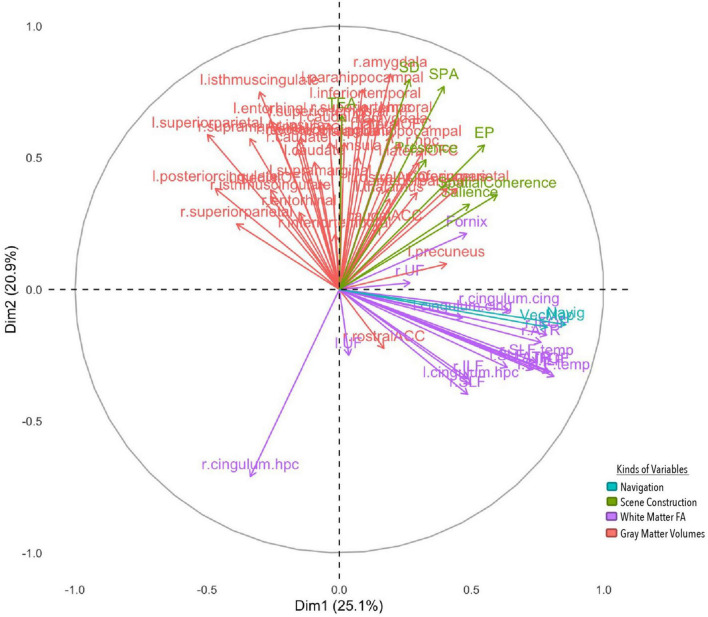
MFA variable contributions. Variables are distinguished by color for whether they are a gray matter volume, measure of white matter FA, or aspect of performance on the navigation or scene construction tasks. Variables that covary together positively point in the same direction on a specific axis, and variables that covary together negatively point in opposite directions.

The first four components were significant and explained a total of 63.4% of the variance, however, we only discuss the first two components in detail (see Supplementary Table 1 for a full list of variables, components and eigenvalues, and Figure 4 for visualization).

The first component explained 25.1% of the total variance, implicating white and gray matter structures with spatial processing broadly. Vector mapping and navigation performance loaded positively onto this component, along with the spatial coherence index and salience measures from the scene construction task. White matter structures also loaded positively, including long association tracts connecting frontal with occipital neocortices [bilaterally the IFOF and superior longitudinal fasciculi (SLF)], and temporal with occipital neocortices [bilaterally the inferior longitudinal fasciculus (ILF)], as well as white matter tracts connecting paralimbic cortices (bilateral cingulate cingulum bundles) and projections forming the extended hippocampal system to the thalamus (fornix) and thalamo-prefrontal connections (anterior thalamic radiation). Gray matter volume for the precuneus also loaded positively, but volume for the superior parietal lobule loaded negatively. Results from this component suggest that those who performed better at spatial processing and integration in both tasks had greater white matter fibre density in cortico-cortical and subcortico-cortical projection including core memory/navigation neural systems, as well as greater high-order visual associative cortices volume (precuneus), but smaller volumes of the superior parietal lobule, often implicated in attentional functions.

The second component explained 20.9% of the total variance, implicating prefrontal, temporal, limbic and paralimbic cortical structures with detail generation. All four detail types in the scene construction task along with subjective presence loaded on to the component positively. Gray matter volumes were also positively loaded bilaterally, which included cortical structures implicated in higher-order reward learning (lateral orbitofrontal cortex, lOFC) and speech/language processing (superior temporal gyrus), limbic structures implicated in associative binding (hippocampus) and emotional processing (amygdala), and paralimbic structures that process scenes (parahippocampus) and connect with cingulate and temporal cortices (isthmus cingulate). Surprisingly, a white matter tract on the right hemisphere connecting the parahippocampus with precuneus and superior parietal lobule (cingulum) negatively contributed to this component. This suggests that those who could imagine more detailed scenes felt more present within them and had greater volume in structures implicated in scene processing and making higher order valued associations, along with lower white FA connecting such structures to those necessary for making higher order visual associations.

**FIGURE 5:**
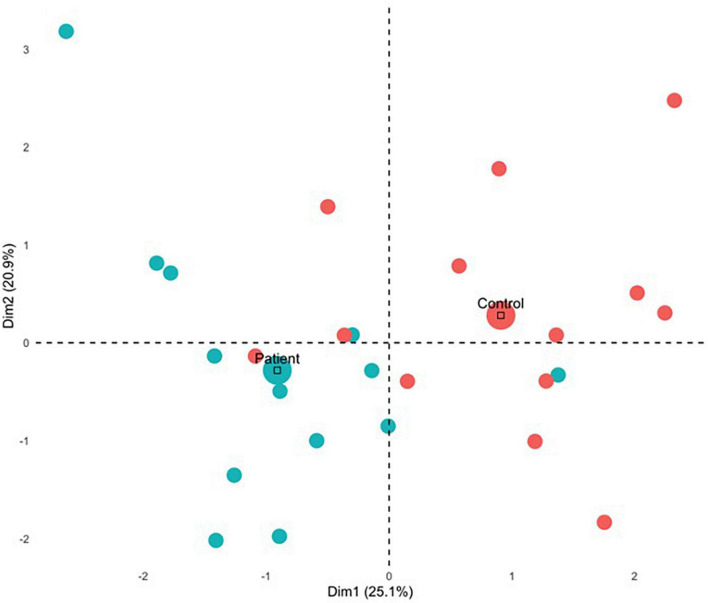
MFA individual contributions. Mean contribution for each group is indicated by the large circle. Control and patient participants contributed differently for Dimension 1 and Dimension 2, as they are in orthogonal quadrants wherein patients tended to contribute negatively and control participants tended to contribute positively.

For the first component, group differences emerged (see Figure 5) with all but 1 PTSD patient showing negative component scores, and all but three controls showing positive scores. This was also reflected in a correlational analysis revealing that participants’ contribution to the first component had a significant negative relationship with CAPS symptoms, *r*_*t*_(24) = −0.46, *p* = 0.001 such that those with a greater symptom severity contributed more negatively to Component 1 (Table 4). The correlation between Component 1 contributions and overall CAPS score was a medium to large effect size, as were the effect sizes for correlations of participants contributions to the first component with their scores on the CAPS subscale of Arousal, Avoidance, and Re-experiencing. Although PTSD symptom severity did not have a statistically significant relationship with individual contributions to the second component, *r*_*t*_(24) = 0.11, *p* = 0.430, there was no significant statistical difference between correlation coefficients, *z*(24) = −1.33, *p* = 0.093.

**TABLE 4:** Correlation between symptom severity and individual contributions.

	Component 1	Component 2
CAPS	−0.46 (*p* = 0.001)	−0.11 (*p* = 0.430)
Arousal	−0.45 (*p* = 0.002)	−0.20 (*p* = 0.176)
Avoidance	−0.55 (*p* < 0.001)	−0.03 (*p* = 0.836)
Re-experiencing	−0.37 (*p* = 0.012)	−0.13 (*p* = 0.395)

## Discussion

Compared to trauma-exposed matched controls, patients with PTSD showed significant impairments in virtual navigation and in scene construction abilities, especially those pertaining to spatial representations. Bootstrapping and permutation tests from the MFA revealed that spatial deficits were associated with abnormal white matter integrity to cortico-cortical long association tracts as well as limbic and paralimbic white matter tracts forming parts of the core memory and navigation neural systems. Reduced detail generation within the imagined scenes was associated with reduced gray matter volumes to frontal and limbic structures, which was mostly driven by patients. Reduced hippocampal volumes were associated with reduced objective and subjective richness of scene construction, but contrary to our prediction, were not major contributors to group differences. However, group differences gave rise to reduced hippocampal white matter connectivity with cortical regions and subcortical structures which were predictive of spatial and scene deficits. These findings, along with how they relate to the clinical phenomenology and neurobiology of PTSD will be discussed in turn below.

We found that patients with PTSD are markedly impaired at neutral scene construction compared to trauma-exposed controls, and on the backdrop of this overall impairment, they imagined proportionally fewer spatial details and more person-related details. Scenes imagined by patients with PTSD were also less vivid, less integrated, and patients felt less present within them, qualities that possibly reflect the spatially fragmented nature of their constructed scenes. Scene construction is thought to underpin the process of mental simulation for both past and future episodic experiences (Schacter et al., 2007), that allow self-projection into alternate realities in the mind’s eye. Those with PTSD provide fewer and less detailed recollections of past non-traumatic experiences than non-clinical controls (Schönfeld et al., 2007; Moradi et al., 2012; Graham et al., 2014; Ono et al., 2016; Schönfeld and Ehlers, 2017). These recollections are more often related to the trauma or to their life beforehand and cause greater rumination (Schönfeld and Ehlers, 2017), a reduced self-reported meaning of life (Waytz et al., 2015), and to a lack of sense of a positive and hopeful future (Feldman and Snyder, 2005). Most interesting is that here, PTSD patients imagined scenes that are non-traumatic, and do not require self-projection either to the past or to the future yet showed similar impairments. Patients’ construction performance was similar to that seen in traumatic memory recollection, showing deficits in both the generation of details and their integration into a greater context (Van der Kolk and Fisler, 1995; Halligan et al., 2003). Our findings suggest that cognitive deficits in PTSD reach beyond memory of the traumatic event itself or even episodic past or future thinking to processes that more broadly underlie episodic memory and other cognitive functions that are not necessarily autobiographical. Future investigations could probe the extent to which these deficits constitute pre-trauma risk factors and their relationship to other cognitive risk factors of PTSD such as IQ, verbal abilities, autobiographical memory, or processing speed (DiGangi et al., 2013).

Scene construction impairment in PTSD patients was as severe as that found originally in hippocampal amnesic patients (Hassabis et al., 2007). In both studies, scenes imagined by patients had a strong deficit in spatial coherence where scenes were fragmented and lacked richness and vividness. Because of previously reported reduced hippocampal volumes in PTSD, and the known hippocampal involvement in spatio-temporal aspects of scene construction, we had predicted hippocampal volumes would be related to deficits in scene construction. However, in the multiple factor analysis hippocampal volumes were not related with spatial processing in either task. Rather, spatial processing deficits were related to abnormal white matter microstructure in tracts that connect the hippocampus, thalamus, and cortical sensory regions. This pattern was mostly driven by patients with PTSD. In conjunction with behavioral findings, this suggests that it is the changes to inter-region communication with the hippocampus that leads to spatial integration deficits, not necessarily hippocampal volume *per se*. In addition, spatial processing deficits were associated with abnormal cortical volume in regions implicated in spatial manipulation (larger superior parietal lobule; Gogos et al., 2010) and intrusive re-experiencing (smaller precuneus; Kitamura et al., 2021). Smaller precuneus volumes have also previously been shown to be associated with reduced spatial aspects of personal memories (Hebscher et al., 2018) and disruption of precuneal activity causes network-wide disruption of the autobiographical recollection network and reduced vividness of retrieved memories (Hebscher et al., 2019, 2020). Thus, the involvement of the precuneus in scene construction is consistent with its role in recall of detail rich personal memories as well.

The second factor in the multiple factor analysis indicated that reduced detail generation in imagined scenes and reduced sense of presence within the scene were related to reduced volumes in limbic structures, including the amygdala and hippocampus. This pattern was not specific to those diagnosed with PTSD, although many of the patients did contribute significantly to this pattern. This is in alignment with extensive literature suggesting the hippocampus is necessary for vivid recollection of detailed episodic experiences, and especially critical for rich re-experiencing (Addis et al., 2004; Gilboa et al., 2004). Imagining less detailed and present scenes was also associated with reduced volume in structures involved in scene processing (i.e., parahippocampus and superior temporal gyrus) and the lOFC, which is involved in contingency mapping and implicated in the neurobiology of intrusive cognitions (Milad and Rauch, 2007). The second factor was also related to increased white matter integrity in the right hippocampal cingulum bundle which is part of the posterior segment of the circuit of Papez, connecting parahippocampal cortices, cingulate and anterior thalamic nuclei. Due to its varied connections, hippocampal cingulum integrity is associated with episodic and emotional processing but also implicated in neurodegeneration pathology (Choo et al., 2010). Increased cingulum FA has been previously reported in this population (Zhang et al., 2012; Kennis et al., 2015) and may be acquired due to the neurotoxic effects of chronic stress (Zhang et al., 2012; Kennis et al., 2015), however, the laterality of FA increases have been inconsistent and decreases have also been found (Schuff et al., 2011; Fani et al., 2012; Bierer et al., 2015). Thus, it is still unclear whether white matter cingulum bundle changes are due to PTSD outcome, however, our results align with the idea that some PTSD symptomatology may be due to reduced gray matter volume but increased connectivity between regions (Mueller et al., 2015).

Our findings are also consistent with the updated dual-representation model of PTSD (Brewin et al., 2010) which accounts for intrusive imagery across psychiatric disorders in terms of healthy voluntary episodic memory processes. This theory posits that the imagery of representations that are flexible, consciously accessible and context-dependent involves different neural structures than inflexible and involuntary sensory-dependent representations. These two forms of imagery converge on representations mediated by the precuneus. Sensory-dependent memories involve regions implicated in imagery (e.g., precuneus), interoception (e.g., insula), exteroception (early sensory regions), and affect (e.g., amygdala) and are more strongly connected in individuals with PTSD, but have weaker communication with other limbic (i.e., hippocampus) and subcortical regions (i.e., thalamus) and regions implicated in spatial processing (i.e., parahippocampus, sensory association areas) or perspective shifting (i.e., retrosplenial and parietal regions) that are involved in context-dependent representations. Even though in our study only neutral scene imagery was voluntarily imagined, we found partial support of this model where patients with PTSD had more fragmented context-dependent scene imagery, which was associated with a pattern of decreased white matter integrity between limbic regions and regions implicated in spatial processing.

Our study has some limitations, such as a lack of accounting for the individuals’ specific trauma history. The etiology of PTSD is complex and can differ based on predisposing factors such as one’s geographical location, gender, and previous trauma history (Kilpatrick et al., 2013), in-the-moment factors such whether the individual dissociated during the traumatic experience (Friedman, 2013), and whether the individual had support after the event. Although the type of trauma and time since the event were relatively uniform across our patients and controls, unfortunately, we were unable to collect history of trauma and reaction to the event information from participants. Our use of individual differences and multivariate approach mitigates some of these shortcomings, although our sample size may be considered small for when considering beyond the first component.

To conclude, in the present study, we found that patients with PTSD show robust impairments in navigation and scene construction processing compared to trauma-exposed controls. Scene construction deficits were seen in qualitative aspects of the scenes and detail generation, but deficits were especially pronounced for spatial aspects of the scenes and the integration of details within this greater context. Thus, scene imagery of neutral scenes in PTSD patients echoes the patterns of mnemonic deficits characteristic of the disorder, such as reduced detail generation and fragmentation, but for a non-traumatic and non-mnemonic based task. Scene construction is thought to be the basis of episodic memory processes; therefore, our findings suggest a generalized cognitive deficit for imagery in patients which aligns with the dual-representation model of PTSD (Brewin et al., 2010). In our multivariate analyses, spatial deficits in patients were found to be related to abnormal white matter integrity to cortico-cortical long association tracts and limbic and paralimbic tracts contributing to memory and navigation neural networks. Detail generation deficits were related to reduced gray matter volumes in frontal and limbic regions, and this pattern was mostly driven by patients. These results are unique as they examine both white and gray matter changes in those who have experienced trauma, and these neural changes were found to be predictive of PTSD symptomatology and behavioral deficits. This speaks to the strength of using multivariate statistical approaches to psychiatric research as one can examine the dynamic relationship between various neural changes, behavior, and group membership. Previous research on PTSD using univariate approaches has found contradictory patterns of neural changes, perhaps because these neural systems are multidimensional, dynamic, and adaptive for the individual. Similar to other psychiatric disorders (Mayberg, 2003), PTSD affects discrete yet integrated pathways that involve select cortical, limbic and paralimbic structures. Conceptualizing PTSD phenomenology at a systems-level of dysfunction will inform our understanding of the various subtypes of PTSD and other trauma disorders, and hopefully lead to appropriate patient-specific treatments.

## Data Availability Statement

The raw data supporting the conclusions of this article will be made available by the authors, without undue reservation.

## Ethics Statement

The studies involving human participants were reviewed and approved by the Institutional Ethics Review Board (RMB-0300-09) at RAMBAM Medical Centre in Haifa. The patients/participants provided their written informed consent to participate in this study.

## Author Contributions

AG designed the experiment. LA-A and SA-L collected the data and scored the behavioral tasks. HM and DB conducted the data analyses. AG and HM wrote the manuscript. All authors contributed to the article and approved the submitted version.

## Conflict of Interest

The authors declare that the research was conducted in the absence of any commercial or financial relationships that could be construed as a potential conflict of interest.

## Publisher’s Note

All claims expressed in this article are solely those of the authors and do not necessarily represent those of their affiliated organizations, or those of the publisher, the editors and the reviewers. Any product that may be evaluated in this article, or claim that may be made by its manufacturer, is not guaranteed or endorsed by the publisher.
