# Supplementary material for: Scene Construction and Spatial Processing in Post-traumatic Stress Disorder
Source: Front Behav Neurosci. 2022 Jun 29;16:888358. doi: 10.3389/fnbeh.2022.888358 (PMC9278269; doi:10.3389/fnbeh.2022.888358)
Supplement: Supplementary file 1 [file Data_Sheet_1.pdf]

Supplementary Material

1 Scene Construction Task

1.1 Subjective Ratings Provided by Participants

Thinking about the scene you just imagined rate your sense of presence on a scale from 1 to 5:

Did not feel like I was there at all				Felt strongly like I was really there
1	2	3	4	5

Thinking about the scene you just imagined rate your sense of perceived salience on a scale from 1 to 5:

I could not really see anything				Extremely salient
1	2	3	4	5

1.2 Spatial Coherence Index Provided by Participants

Thinking about the scene you just imagined, here is a list of phrases. Please read each of them and then tick the ones you think best describe your imagined scene, and your experience of trying to imagine it. Pick as many as you think are relevant to you.

1. It was quite fragmented
2. I saw the scene in color
3. It was similar to looking at a picture or seeing it on TV
4. I could see individual details, but it didn't all fit together as a whole scene
5. I would find it easy to answer questions about the scene
6. It wasn't so much a scene as a collection of images
7. I was able to use some senses other than vision (e.g., sound, smell)

8. I could see it as one whole scene in my mind's eye

9. I was able to think of details associated with the general theme

10. I would find it easy to give further details of the surroundings in the scene

11. It wasn't a scene you could step into; it wasn't really joined-up

12. I would find it easy to substitute an aspect of the scene for something else

1.3 Scoring Samples by External Scorer

PTSD201:

SD SD SD EP SR Editorial
Beautiful / large / wide/ stairs / as you enter the museum/. Every museum has its own
SD SR EP EP SD EP
atmosphere /. A large / central/ hallway /. Speech / becomes quieter/. You can see groups /
TEA EP EP TEA TEA TEA EP
that walk around / with guides/. Single people / that stop / and look / and read / the signs /
EP SD SD EP SD EP
on each artifact /. Beautiful building / large / windows / nice / lighting /. (Probe: any other
Editorial Editorial
details?). People invest a lot in artwork/. These are people who dedicated their lives to art /.

Control205:

SD TEA Editorial SD
It is a small / and pleasant coffee shop/. Not one of those disgusting large chain places/. Small/
SD EP SD SD EP EP SD SD TEA
round / tables/ and wine-red / padded / chairs /. Waiters / in black / tricot shirts / walk around
EP TEA SD SD SR
/ with trays / and take orders /. There is a good smell / of brewed coffee / in the air / and the
SD SD EP
Smell of baked goods /. The occasional noise / of the espresso machine / interrupts the
TEA SD EP TEA
Conversation /. It is generally too noisy / because many people / are talking / and there is
SD SD TEA SD EP SD
background music /. They play 90's music /. I drink / a strong / Latte /. It is quite good /
TEA SD TEA EP SD SR
but they added / too much foam /. I eat / an apple pie / with white sugar powder / on top / and
SD EP TEA
it gets on my shirt /. I am with a friend / and we are talking /. (probe: any other details?)/
EP SR TEA SR SD EP
There is a terrace / across / and you can sit / outside /. But it is not quite a terrace / just tables
SR TEA SR TEA EP
/ on the sidewalk / so people walk / right by your table / and that is not pleasant /. The only
TEA SR EP SD
people sitting /outside / are smokers / because it is quite a cold day.

1.4 Quality Ratings by External Scorer

On a scale of 0-10, how well did you feel the description evoked a detailed picture of the experience in your own mind:

No picture at all — **Vivid, extremely rich picture**

0	1	2	3	4	5	6	7	8	9	10

2 Multivariate Analyses

2.1 **Table S1**. **Full list of MFA variables, components, and eigenvalues.** Variables that significantly contribute to each component are in bold.

Variables	***Dimension 1***	***Dimension 2***	***Dimension 3***	***Dimension 4***
	(25.1%)	***(20.9%)***	***(9.1%)***	***(8.3%)***
Gray Matter Volumes				
Right Lateral OFC	*0.92*	***2.98***	*0.42*	*1.27*
Right Medial OFC	*-0.54*	*1.69*	*-0.66*	*1.91*
Right Caudal ACC	*0.67*	*1.17*	*0.19*	*0.90*
Right Rostral ACC	*0.65*	*-0.77*	*-0.12*	*-1.58*
Right Isthmus Cingulate	*-1.07*	***2.12***	*-1.05*	*-1.66*
Right Posterior Cingulate	*-0.14*	*1.77*	*-0.84*	*0.95*
Right Precuneus	***2.60***	***2.03***	*-0.90*	*0.02*
Right Inferior Parietal	*1.88*	*1.42*	*-0.56*	*0.43*

Right Inferior Temporal	*-0.07*	*1.02*	*0.51*	***2.37***
Right Superior Parietal	***-2.26***	*1.61*	*1.17*	*1.05*
Right Superior Temporal	*0.60*	***3.52***	*0.03*	*1.33*
Right Supramarginal	*-1.02*	*1.37*	*-0.88*	***2.43***
Right Parahippocampal	*1.29*	***3.01***	*0.82*	*0.20*
Right Entorhinal	*-0.82*	*1.88*	*1.72*	*1.77*
Right Hippocampus	*1.35*	***2.66***	*1.75*	*1.86*
Right Insula	*-0.53*	*1.63*	***-2.08***	***2.64***
Right Amygdala	*0.65*	***2.54***	*1.55*	*1.72*
Right Caudate	*-0.66*	*1.83*	*1.20*	*1.79*
Right Thalamus	*0.05*	***2.90***	*-1.56*	*1.56*
Left Lateral OFC	*1.57*	***2.58***	*0.57*	*1.73*
Left Medial OFC	*-1.38*	*1.34*	*0.63*	*-0.22*
Left Caudal ACC	*0.00*	***2.15***	*0.68*	*1.85*
Left Rostral ACC	*0.97*	*1.94*	*0.29*	*1.28*
Left Isthmus Cingulate	*-1.00*	*1.95*	*0.80*	*0.12*
Left Posterior Cingulate	*-1.86*	*1.22*	*-1.29*	*0.33*

Left Precuneus	*1.87*	*0.36*	*-0.08*	*0.51*
Left Inferior Parietal	*1.43*	*1.88*	*0.18*	***2.28***
Left Inferior Temporal	*0.34*	***2.55***	*-0.81*	*1.92*
Left Superior Parietal	*-1.86*	*1.81*	*0.87*	*1.21*
Left Superior Temporal	*-0.23*	***3.59***	*-1.32*	*0.35*
Left Supramarginal	*-0.39*	***2.40***	*-1.58*	*0.39*
Left Parahippocampal	*0.28*	***2.41***	*0.29*	*0.92*
Left Entorhinal	*-1.05*	***2.40***	*-1.62*	*0.47*
Left Hippocampus	*0.88*	***2.57***	***2.53***	*1.65*
Left Insula	*0.24*	*1.97*	*-1.95*	***3.17***
Left Amygdala	*0.68*	***2.01***	***2.71***	*1.82*
Left Caudate	*-0.40*	*1.76*	*0.62*	***2.72***
Left Thalamus	*1.00*	*1.36*	*-0.33*	*1.43*
White matter FA's				
Right UF	*1.65*	*0.10*	*1.27*	*0.96*
Right ILF	***2.56***	*-1.81*	***3.38***	*0.83*
Right SLF temporal	***3.56***	*-1.23*	***2.36***	*0.54*

Right SLF	***2.50***	***-2.17***	***3.02***	*1.40*
Right Cingulum HPC	*-1.85*	***-3.11***	*1.77*	*1.08*
Right Cingulum Cingulate	***4.41***	*-0.50*	*1.13*	*0.45*
Right ATR	***3.99***	*-0.92*	***2.48***	*1.00*
Right IFOF	***3.93***	*-0.69*	***2.55***	*-0.14*
Fornix	***3.27***	*1.03*	*-0.36*	*-0.46*
Left UF	*0.27*	*-1.45*	***2.35***	*0.50*
Left ILF	***3.57***	*-1.27*	***2.57***	*-0.37*
Left SLF	***3.26***	*-1.84*	***2.77***	*1.00*
Left SLF Temporal	***3.78***	*-1.36*	***2.18***	*0.32*
Left Cingulum HPC	***2.43***	*-1.52*	*-0.02*	*1.48*
Left Cingulum Cingulate	***3.22***	*-0.99*	*1.85*	*0.87*
Left ATR	***3.51***	*-1.36*	***2.12***	*0.68*
Left IFOF	***3.60***	*-1.24*	***2.50***	*-0.21*
Scene Construction Performance				
Presence	*1.97*	***2.31***	*-0.04*	***-2.89***
Salience	***2.84***	*1.61*	*0.24*	***-2.80***

Spatial Coherence	***3.59***	*1.87*	*-0.76*	***-2.37***
Spatial References	***2.06***	***3.60***	*0.25*	*-0.94*
Entities Present	***3.10***	***2.68***	*0.99*	*-1.98*
Sensory Descriptions	*1.23*	***3.27***	*0.60*	*-1.23*
Thoughts, Emotions, Actions	*0.03*	***2.85***	***2.22***	*0.08*
VR Navigation Performance				
Navigation	***4.60***	*-0.58*	*-1.79*	*0.61*
Vector Mapping	***4.74***	*-0.70*	***-2.05***	*1.02*

Note: OFC: orbitofrontal cortex; ACC: anterior cingulate cortex; UF: uncinate fasciculus; ILF: inferior longitudinal fasciculus; SLF: superior longitudinal fasciculus; HPC: hippocampus; ATR: anterior thalamic radiation; IFOF: inferior frontal-occipital fasciculus

Blue: measures from the navigation task

Green: measures from the scene construction task

Purple: white matter tracts

Pink: grey matter volumes

2.2 Discussion of Components 3 and 4

The third component explained 9.1% of the shared variance. Those who reported a greater number of person related details in their scenes had increased white matter FA bilaterally to the ATR, SLF, ILF, IFOF and had increased volume to the left amygdala and left hippocampus. The fourth component explained 8.3% of the shared variance, showing those who imagined scenes with reduced quality (i.e., reduced presence, salience, and spatial coherence) had increased grey matter volume to the right inferior temporal gyrus, right supramarginal gyrus, left inferior parietal gyrus, left caudate, and insula bilaterally.

PTSD symptom severity did not have a statistically significant relationship with individual contributions to the third, $r_t = .10$, $p = .513$, or fourth components, $r_t = .10$, $p = .513$. This is especially interesting when contrasting the results of component one, which differentiated patients, and component three, which did not, although correlation coefficients were not significantly different, $z = -1.31$, $p = .095$. In component one, patients showed reduced spatial processing along with reduced white matter FA to long association white matter tracts. In component three, those who reported more personal details show increased FA to those same long association tracts, irrespective of symptom severity.
